# Distinct genomic subclasses of high-grade/progressive meningiomas: NF2-associated, NF2-exclusive, and NF2-agnostic

**DOI:** 10.1186/s40478-020-01040-2

**Published:** 2020-10-21

**Authors:** Erik A. Williams, Sandro Santagata, Hiroaki Wakimoto, Ganesh M. Shankar, Fred G. Barker, Radwa Sharaf, Abhinav Reddy, Phoebe Spear, Brian M. Alexander, Jeffrey S. Ross, Priscilla K. Brastianos, Daniel P. Cahill, Shakti H. Ramkissoon, Tareq A. Juratli

**Affiliations:** 1 Foundation Medicine Inc, 150 Second Street, Cambridge, MA 02141 USA; 2 Translational Neuro-Oncology Laboratory, Department of Neurosurgery, Massachusetts General Hospital Cancer Center, Harvard Medical School, Boston, MA USA; 3 Department of Pathology, Brigham and Women’s Hospital, Boston, MA USA; 4 Department of Neurosurgery, Massachusetts General Hospital Cancer Center, Harvard Medical School, Boston, MA USA; 5 Department of Pathology, State University of New York Upstate Medical University, Syracuse, NY USA; 6 Stephen E. and Catherine Pappas Center for Neuro-Oncology, Division of Hematology/Oncology, Department of Neurology, Massachusetts General Hospital, Harvard Medical School, Boston, MA USA; 7 Department of Pathology, Wake Forest School of Medicine, Wake Forest Comprehensive Cancer Center, Winston-Salem, NC USA; 8 Department of Neurosurgery, University Hospital Carl Gustav Carus, Technische Universität Dresden, Fetscherstr. 74, Dresden, Germany

**Keywords:** Meningioma, 1p loss, Progressive, NF2, TERT, PBRM1, CDKN2A, KDM6A, Metastatic

## Abstract

**Background:**

Genomic studies of high-grade/progressive meningiomas have reported a heterogeneous mutation spectrum, identifying few recurrently mutated genes. Most studies have been underpowered to detect genomic subclasses of aggressive meningiomas due to relatively small number of available samples. Here, we present a genomic survey of one of the largest multi-institutional cohorts of high-grade/progressive meningiomas to date.

**Methods:**

850 high-grade/progressive meningiomas, including 441 WHO grade 2 and 176 WHO grade 3 meningiomas and 220 progressive WHO grade 1 meningiomas, were tested as part of a clinical testing program by hybridization capture of 406 cancer-related genes to detect base substitutions, indels, amplifications, deletions, and rearrangements. Information from pathology reports, histopathology review, and patient clinical data was assessed.

**Results:**

Genomic analyses converged to identify at least three distinct patterns of biologically-aggressive meningiomas. The first and most common contained *NF2*-mutant tumors (n = 426, 50%), was associated with male sex (64.4% %, *p* = 0.0001) and often harbored additional mutations in *CDKN2A*/*B* (24%), and the chromatin regulators *ARID1A* (9%), and *KDM6A* (6%). A second group (*NF2*-agnostic) featured *TERT* promoter (*TERT*p; n = 56) or *TP53* mutations (n = 25) and were either *NF2*-mutant or wild-type, and displayed no association with either sex (*p* = 0.39). The remaining group generally lacked *NF2* mutations, and accounted for 40% of the cases—with three subgroups. One consistent primarily of grade 3 lesions harboring alterations in chromatin regulators *BAP1* (n = 22) or *PBRM1* (n = 16). A second subgroup contained *AKT1* (n = 26), *PIK3CA* (n = 14) and *SMO* (n = 7) mutant skull-based meningiomas, and a third mixed subgroup included 237 meningiomas with a heterogeneous spectrum of low frequency and non-recurrent alterations.

**Conclusions:**

Our findings indicate that the patterns of genomic alterations in high-grade/progressive meningiomas commonly group into three different categories. The most common *NF2*-associated canonical group frequently harbored *CDKN2A*/*B* alterations, which is potentially amenable to targeted therapies. An *NF2*-agnostic group harbored frequent *TERT*p and *TP53* mutations. The final subclass, distinct from the canonical *NF2* mutant associated pathway, was partly characterized by *BAP1*/*PBRM1* alterations (rhabdoid/papillary histology) or skull-base disease. Overall, these data increase our understanding of the pathobiology of high-grade/progressive meningiomas and can guide the design of clinical trials.

**IRB approval status:**

Reviewed and approved by Western IRB; Protocol No. 20152817.

**Electronic supplementary material:**

The online version of this article (10.1186/s40478-020-01040-2) contains supplementary material, which is available to authorized users.

## Introduction

Our understanding of the molecular biology of meningiomas has significantly improved in the last decade. This new era began with the publication of two landmark genetic sequencing studies in 2013, in which *AKT1*, *SMO*, *TRAF7* and *KLF4* were identified as frequently mutated genes in skull base, WHO grade 1 meningiomas [5, 6]. Since that time, much work has been carried out to detect additional recurrently mutated genes involved in the molecular pathogenesis of these tumors [1, 7, 37].

WHO grade 2 and 3 meningiomas have high-grade histologic features and represent up to 25% of all meningiomas. Affected patients frequently experience disease progression despite treatment, with aggressive regrowth resulting in high morbidity and mortality. There is substantial heterogeneity in clinical presentation amongst these tumors, as some initially present as benign grade 1 disease, and only manifest biologically-aggressive progression after many years (either as recurrent grade 1, or transformed higher-grade disease), while other tumors are higher grade (2 or 3) at initial presentation. Investigators have long sought effective predictors of meningioma recurrence and malignant transformation, to guide the administration of intensified treatments such as radiation or radical surgery, or to identify genomic subsets that are amenable to targeted therapies.

The genetic profiles of high-grade meningiomas are dominated by inactivation of the *NF2* tumor suppressor gene. However, more data have accumulated regarding alterations in genes not previously implicated in meningiomas that appear to delineate subsets of progressive meningiomas with unfavorable clinical prognoses. These aberrations include promoter mutations and rearrangements of *TERT*, and inactivating mutations in *BAP1*, *PBRM1*, *ARID1A*, and *SMARCE1* as well as focal deletions of the dystrophin gene (*DMD*) [2, 3, 14, 19–21, 28, 33, 41]. Mutations in some of these genes are enriched in tumors with distinct histologic features such as *SMARCE1* in clear cell meningioma, and *BAP1* and *PBRM1* in rhabdoid and papillary meningiomas, respectively [33, 36, 41].

Despite this progress in identifying recurrently mutated genes in aggressive meningiomas, these genes are altered in fewer than 35% of high-grade/progressive meningiomas, suggesting the existence of additional aberrations in unidentified or underrepresented cancer genes. Notably, most genomic studies of high-grade meningiomas have been underpowered to detect recurrent clinically actionable mutations and distinct high-grade meningioma molecular subclasses. Consequently, most studies have reported a heterogeneous spectrum of mutations with few recurrently mutated genes [27, 28, 32] and have not provided detailed information regarding how often these mutations co-occur with one another.

In this study, we performed comprehensive genomic profiling of one of the largest sets of WHO grades 2 and 3 meningiomas analyzed to date. We also included analysis of clinically-progressive grade 1 meningiomas. We aimed to assess clinically actionable mutations to facilitate identifying patients amenable to targeted treatment trials. In addition, we sought to improve our understanding of genomic events underlying high-grade/progressive meningiomas.

## Methods

### Tumor samples and molecular genetic analysis

Samples from 850 meningiomas were analyzed as part of a clinical care for patients using comprehensive genomic profiling (CGP) in a Clinical Laboratory Improvement Amendments-certified, College of American Pathologists-accredited laboratory (Foundation Medicine, Cambridge, MA). Approval for this study, including a waiver of informed consent and a HIPAA waiver of authorization, was obtained from the Western Institutional Review Board (Protocol No. 20152817). The pathologic diagnosis of each case was first made in the referring center and was then confirmed in our facility (Foundation Medicine, Cambridge, MA) on routine hematoxylin and eosin stained slides. All samples that contained a minimum of 20% tumor nuclear nuclei were forwarded for DNA and/or RNA extraction. WHO grade was not available for a small subset of samples (n = 13).

Technical descriptions and validation of the genomic profiling assays used to analyze these samples in the course of clinical care have been published previously [12, 16]. In brief, ≥ 50 ng DNA was extracted from 40 µm scrolls from formalin-fixed, paraffin-embedded tissue blocks of tumor. The samples were assayed by adaptor ligation hybrid capture, performed for all coding exons of 236 (v1), 315 (v2), or 405 (v3) cancer-related genes plus select introns from 19 (v1), 28 (v2), or 31 (v3) genes frequently rearranged in cancer (Additional file 1: Table S1) [12, 16]. For those samples for which RNA was available, targeted RNA-seq. was performed for rearrangement analysis in 265 genes [20]. RNA sequences were analyzed for the presence of rearrangements only. Sequencing of captured libraries was performed using an Illumina technology to a mean exon coverage depth of 593 ×, and resultant sequences were analyzed for base substitutions, insertions, deletions, copy number alterations (focal amplifications and homozygous deletions), and select gene fusions, as previously described [12, 16]. Clinically relevant genomic alterations (CRGA) were defined as alterations that are targetable by anticancer drugs currently available on the market or in registered clinical trials. Germline variants documented in the dbSNP database (dbSNP142; http://www.ncbi.nlm.nih.gov/SNP/), with two or more counts in the ExAC database (http://exac.broadinstitute.org/), or recurrent variants of unknown significance that were predicted by an internally developed algorithm to be germline were removed, with the exception of known driver germline events (e.g., documented hereditary *BRCA1*/*2* and deleterious *TP53* mutations). Known confirmed somatic alterations deposited in the Catalog of Somatic Mutations in Cancer were highlighted as biologically significant [11]. All inactivating events (i.e., truncations and deletions) in known tumor suppressor genes were also called as significant. To maximize mutation-detection accuracy (sensitivity and specificity) in impure clinical specimens, the test was previously optimized and validated to detect base substitutions at a ≥ 5% mutant allele frequency (MAF), indels with a ≥ 10% MAF with ≥ 99% accuracy, and fusions occurring within baited introns/exons with > 99% sensitivity [12].

### Molecular assignment of subclasses

Based upon mutational phenotype in each sample, we assigned the tumors to at least three subclasses. The used molecular assignment relies on the growing body of literature of molecular patterns that have been recently described in meningiomas [25, 32, 42, 43].

### Statistical analysis

The statistical association of detected somatic alterations with other factors, including age, sex, and tumor location were analyzed using the Fisher exact and Mann–Whitney-U tests. Cases with unavailable molecular or histology data were excluded from the final correlation analysis. A two-tailed *p* value of < 0.05 was considered to be statistically significant. Furthermore, R statistics system version 3.6.1 (https://www.r-project.org/) together with the UpSetR library [8] was used to construct the diagrams of set intersections used in this paper.

## Results

### Patient demographics

We identified 850 biologically-aggressive meningiomas (defined further below) with available molecular data that were profiled in the comprehensive genomic profiling (CGP) program at Foundation Medicine between 2013 and 2019. These tumors were resected from different patients; 466 females and 384 males (ratio 1.2:1). The median patient age was 57 years (range 0–89 + years). While the cohort included meningiomas of all WHO grades (1, 2 and 3), it predominantly consisted of high-grade meningiomas (441 WHO grade 2 and 176 WHO grade 3), in addition to 220 “progressive” WHO grade 1 meningioma defined as relapsed or “under-treatment” progressive tumors. Tumor locations included the skull base (n = 243), supratentorial area (non-skull base, n = 430), non-CNS sites including the skin of the head and distant metastases (n = 32), and spine (n = 30; three of these spine meningiomas were clinically considered as likely drop metastases). The data about tumor location was not available for 114 cases. (Additional file 1: Table S1).

### Genomic alterations

**Fig. 1:**
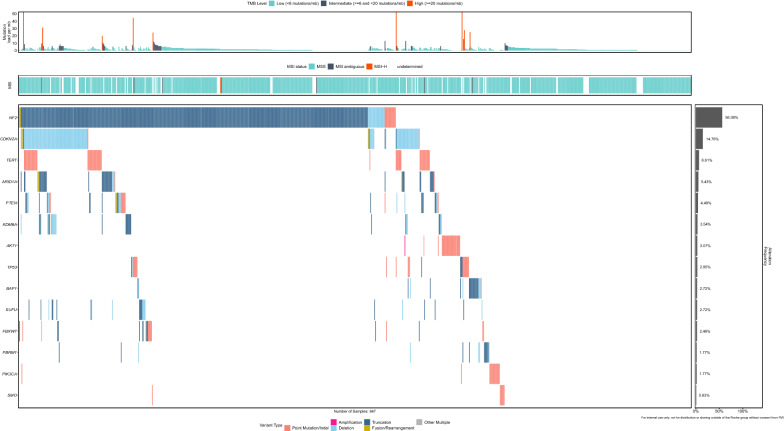
Tile plot to summarize molecular alterations in progressive/high-grade meningiomas

**Fig. 2:**
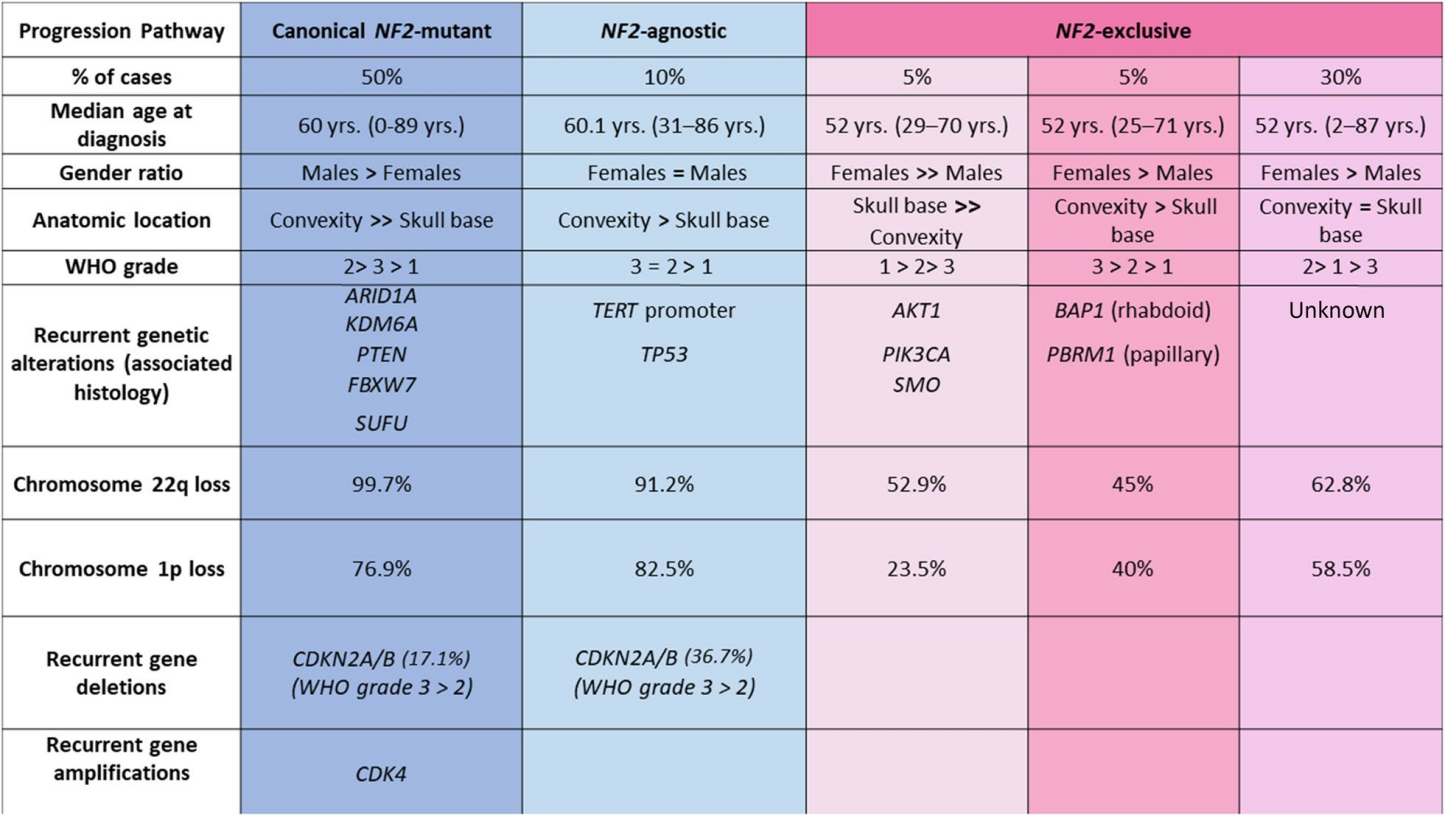
Comparison of three distinct patterns of biologically-aggressive meningiomas. The first and most common subclass (50%) contained *NF2*-mutant tumors, was associated with male sex and harbored additional alterations in *CDKN2A*/*B*, *ARID1A*, *PTEN*, and *KDM6A*. A second group featured *TERT*p or *TP53* mutations and were either *NF2*-mutant or wild-type and with no association with either sex. The remaining group which accounted for 40% of the cases, generally lacked *NF2* mutations and harbored alterations in *BAP1*/*PBRM1*, with a further subgroup containing *AKT1*, *PIK3CA* and *SMO* mutant meningiomas and a third with a heterogeneous spectrum of low frequency and non-recurrent alterations

Loss of chromosome 22q (84.1% of eligible cases) and loss of chromosome 1p (68.8%) were the most common copy number alterations in our cohort (Additional file 2: Figure S1). *NF2* mutations represented the most frequent gene mutations (n = 474/850, 55.7%). While *NF2* mutations and 22q loss events were correlated, there were several cases with 22q loss without detectable *NF2* mutation, raising the possibility of cryptic *NF2* inactivation. The frequency of *NF2* mutations increased significantly with the WHO grade: 81/220 of grade 1 (36.8%), 265/441 of grade 2 (60.1%, *p* = 0.0001) and 122/176 of grade 3 (69.3%, *p* = 0.0001) meningiomas harbored *NF2* mutations. Moreover, male patients (n = 245/384, 64%) were significantly more affected by *NF2*-mutant meningiomas than female patients (n = 227/466, 48.7%, *p* = 0.0001). We detected frequent genomic alterations of genes encoding regulators of the cyclin-dependent kinase inhibitor pathway, including *CDKN2A*/*B* deletions (n = 123/850, 14.4%), *CDKN2C* mutations (n = 16/850, 1.9%), and *CDK4* amplifications (17/850 cases with high level amplifications ≥ 4 gene copies, 2%). Additional frequently altered genes included: *TERT* (7.1%, 56 promoter hotspot mutations of 789 sequenced cases), *ARID1A* (5.4%, 46 cases, including one gene structural translocation), *PTEN* (4.3%, 27 mutations, 10 focal gene deletions and one *PTEN_TMEM38A*_rearrangement), *KDM6A* (3.5%; 30 cases, including focal exon deletions), *SUFU* (2.7%, 17 mutations, 5 focal exon deletions and one gene rearrangement), *TP53* (2.9%, 23 mutations, 1 focal deletion and 1 gene translocation), *BAP1* (2.7%, 18 mutations and 5 exon deletions) and *PBRM1* (1.8%, 10 mutations and 5 focal exon deletions). This comprehensive genomic characterization suggested that high-grade/progressive meningiomas could be separate into largely distinct classes; for further analysis, we divided the tumors into three overall subclasses (Figs. 1, 2). All genomic data are shown in Additional file 1: Table S1.

### The *NF2*-associated pathway of meningiomas (the canonical pathway)

This group included 426 meningiomas (50.1%), and represented the largest group of tumors. The *NF2* alterations involved 393 *NF2* missense mutations (93%), 21 two copy deletions (4.5%) and 12 *NF2* structural rearrangements (2.5%). The vast majority of tumors (99.7%) showed synchronous chromosome 22q LOH and 76.9% showed a 1p chromosome loss. The median age of patients was 59 years (range 0–89 + years). We also observed an enrichment in alterations in *CDKN2A*/*B*, *KDM6A*, *ARID1A*, *PTEN*, *FBXW7*, and *SUFU* in comparison with *NF2*-wt meningiomas (Additional file 3: Figure S2).

Bi-allelic *CDKN2A*/*B* deletions occurred in 93/472 of all *NF2*-mutant (19.7%) versus 30/377 in *NF2*-wt meningiomas (8.0%) (*p* = 0.0001). In the canonical *NF2*-mutant group, 17.1% (n = 72/426) harbored a *CDKN2A*/*B* deletion (Fig. 2). Interestingly, meningiomas harboring *CDKN2A*/*B* alterations were significantly more common in males (n = 77/123, 62.6%) than in females (*p* = 0.0001). *CDKN2A*/*B* alterations were significantly enriched in WHO grade 3 meningiomas (n = 75/176, 42.6%) in comparison to WHO grade 2 (n = 47/441, 10.6%, *p* = 0.0001). Only one WHO grade 1 meningioma had a *CDKN2A*/*B* alteration (n = 1/220, 0.4%, *p* = 0.0001).

**Fig. 3:**
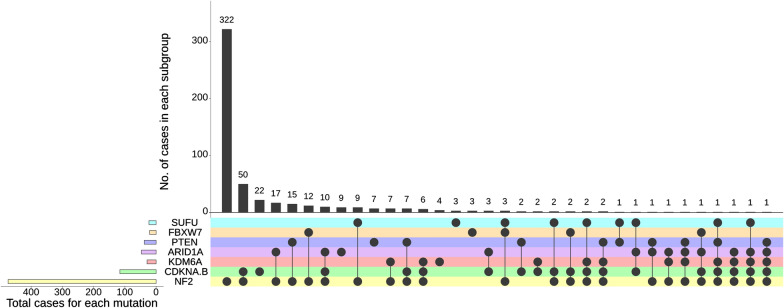
Diagram of set intersection between the most frequently detected genomic alterations in the *NF2*-associated subclass in meningiomas

Furthermore, were observed a significant association of alterations in the chromatin regulator *ARID1A* in *NF2*-mutant meningiomas (n = 34/472, 7%) versus *NF2*-wt meningiomas (n = 12/377, 3.2%, *p* = 0.0138). *ARID1A* alterations were significantly more common in WHO grade 3 meningiomas (n = 26/176, 14.8%) than in WHO grade 2 (n = 18/441, 4.1%, *p* = 0.0001) and WHO grade 1 meningiomas (n = 1/220, 0.4%, *p* = 0.0001). *ARID1A* alterations were significantly more frequently seen in male patients (29/46, 63%, *p* = 0.0146). Moreover, a mutual co-occurrence of *ARID1A* mutations was detected in a subset of meningiomas that harbored aberrations of *CDKN2A*/B (n = 18), *KDM6A* (n = 16), *PTEN* (n = 13), *FBXW7* (n = 3) and *SUFU* (n = 7), as shown in Fig. 3.

*PTEN* mutations were identified in 28/472 of all *NF2*-mutant (5.9%) and in 10/376 of *NF2*-wt meningiomas (2.6%) (*p* = 0.0287). Additionally, *PTEN* mutations were found to be more frequently associated with a WHO grade 3 (n = 15/176, 8.5%) than grade 2 (n = 17/441, 3.8%, *p* = 0.025) or grade 1 (5/220, 2.3%, *p* = 0.0094) meningioma in our cohort. Notably, *PTEN* mutations occurred mutually concurrent with *CDKN2A*/B (n = 13), *KDM6A* (n = 5) and *SUFU* aberrations (n = 2), as shown in Fig. 3.

Importantly, we detected *KDM6A* alterations in 23/472 of all *NF2*-mutant (4.9%) versus 7/377 in *NF2*-wt meningiomas (1.9%) (*p* = 0.0234). Similarly, *KDM6A* alterations occurred significantly more often in WHO grade 3 meningiomas (n = 15/176, 9%) versus 13/441 (2.9%, *p* = 0.0046) and 2/220 (0.9%, *p* = 0.0001) in WHO grade 2 and 1 meningiomas, respectively.

In addition, the meningiomas of 23 patients showed *SUFU* mutations, 18 in *NF2*-mutant (3.7%) and 5 in *NF2*-wt meningiomas (1.3%, *p* = 0.033). *SUFU* mutations were found more frequently in WHO grade 3 (n = 11/176, 6.3%) than grade 2 (n = 11/441, 2.5%, *p* = 0.030) or grade 1 meningiomas (2/220, 0.9%, *p* = 0.0036) in our cohort.

*FBXW7* mutations were present in 23/472 of all *NF2*-mutant (4.9%) and in 3/377 of *NF2*-wt meningiomas (0.8%) (*p* = 0.0004). No significant difference was identified between grades. Interestingly, we observed that *FBXW7* mutations were mutually exclusive to *PTEN* or *KDM6A* mutations (Fig. 3).

Finally, in contrast to *NF2*, *CDKN2A*/*B*, and *ARID1A* alterations, male sex was not significantly associated with gene alterations in *KDM6A*, *PTEN*, *FBXW7* or *SUFU*.

### The *NF2*-agnostic group of meningiomas

This group (n = 79, 9.3%) was characterized by *TERT*p and *TP53* mutations. The median age of patients was 60.1 years (range 31–86 years). *TERT*p mutations occurred in 35/441 (8.1%) of *NF2*-mutant meningiomas and in 21/348 of *NF2*-wt patients (5.7%, *p* = 0.21). WHO grade 3 meningiomas harbored the highest percentage of *TERT*p mutations (23/159, 14.5%), followed by WHO grade 2 (n = 28/421, 6.7%, *p* = 0.0048) and WHO grade 1 meningiomas (n = 5/199, 2.5%, *p* = 0.0001, Additional file 3: Figure S2).

**Fig. 4:**
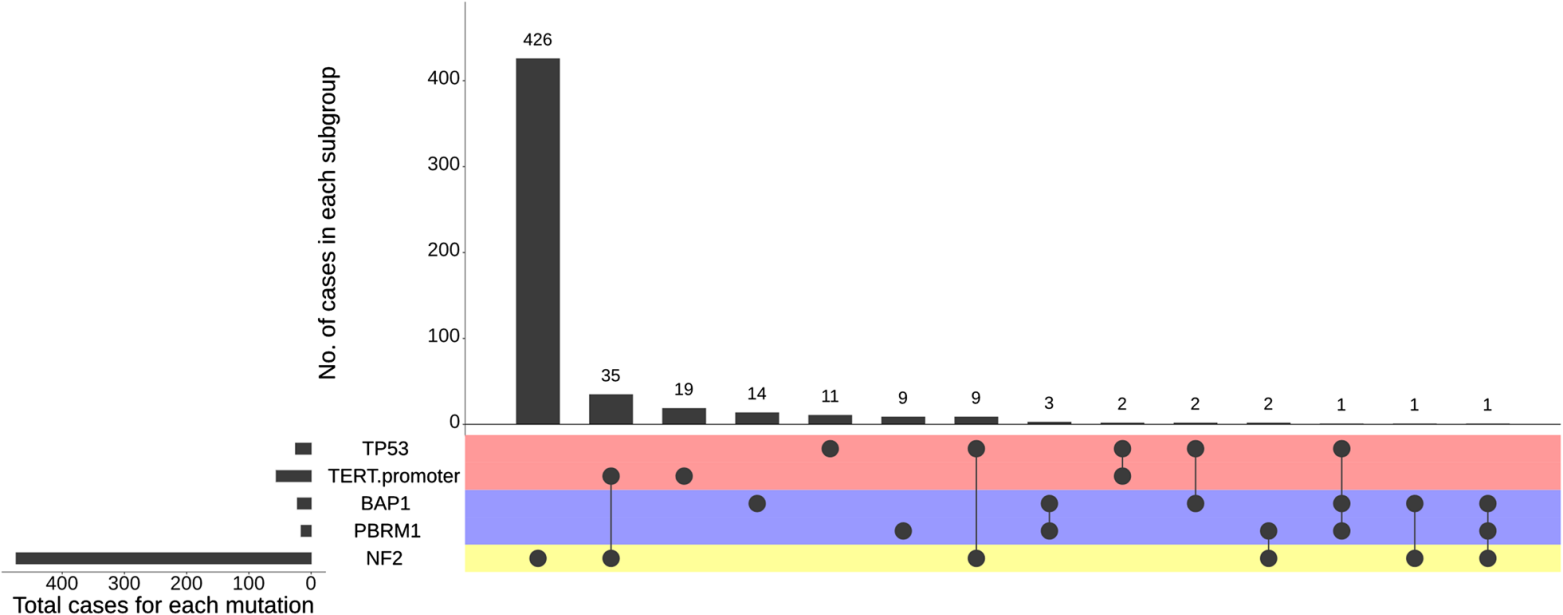
Diagram of set intersection between the most frequently detected genomic alterations in the *NF2*-agnostic subclass in meningiomas

Likewise, *TP53* hotspot mutations were detected in 9/472 *NF2*-mutant meningiomas (1.9%), compared with 16/373 *NF2*-wt meningiomas (4.2%, *p* = 0.063). *TP53* mutations occurred mainly in WHO grade 3 meningiomas (n = 12/176, 6.8%) followed by WHO grade 2 (n = 10/441, 2.2%, *p* = 0.0135) and a significantly lower percentage in grade 1 (n = 2/220, 0.9%, *p* = 0.0018) meningiomas. Notably, two meningiomas harbored simultaneous *TERT*p and *TP53* mutations (Fig. 4). Neither *TERT*p (25 females, 31 males) nor *TP53* (13 females, 12 males) mutations were significantly associated with patient sex. Further frequent genomic alterations in this group were *CDKN2A*/*B* deletions (n = 29/79, 36.7%), chromosome 22q loss (91.2%) and chromosome 1p loss (82.5%), Fig. 2).

### The *NF2* exclusive group of meningiomas contains three subclasses

There were several mutations identified in this third group of high-grade/progressive meningiomas which we further split into three largely distinct subclasses. In one of these subclasses, we found frequent *BAP1* (n = 22) and *PBRM1* (n = 16) alterations—including 5 cases with *BAP1* and *PBRM1* aberrations in the same tumor (total n = 33 tumors, Fig. 4). In addition, 81% of *BAP1* and 87.5% *PBRM1* mutant meningiomas were classified as WHO grades 2 and 3 meningiomas. Simultaneous *NF2* mutations were very infrequently detected (n = 4/33 cases) in this subgroup. Likewise, only 45% and 40% of the meningiomas in this group harbored chromosome 22q and 1p loss, respectively. The median age of patients was 52 years (range 25–76 years). The hallmark of this subgroup is the substantial enrichment for rhabdoid and papillary histology and the not infrequent co-occurrence of these histologic features in the same tumor resection. As previously reported, 11 of 16 *PBRM*1 mutations (68.7%) occurred in meningioma with papillary histologic features [41].

A second subclass within this group contained *AKT1* (n = 26), *PIK3CA* (n = 14) and *SMO* (n = 6) mutant meningiomas (total n = 46, Additional file 2: Figure S1). We observed a marked predominance of female patients in this subgroup (37 females vs. 9 males). In addition, the majority of these *NF2*-wt tumors were classified as WHO grade 1 meningioma (n = 31, 70.4%) and were located almost exclusively in the skull base (Fig. 2). Notwithstanding, this subclass also contained 14 WHO grade 2 meningiomas (8 *AKT1*, 5 *PIK3CA*, and 1 *SMO* case), and a subset of these higher grade cases harbored additional genomic alterations such as *TERT*p mutation (one *AKT1*-mutant meningioma), *TP53* mutation (one *PIK3CA*-mutant case), *PTEN* alterations (2 meningiomas with an *AKT1* mutation), and *CDKN2A* 2 copy loss (1 *AKT1* case; 1 *PIK3CA* case). This finding suggests, due to anatomic location, a more biological aggressive course can occur during progression in these meningiomas that are largely regarded as “benign” and that the presence of these mutations is not invariably indicative of a WHO grade 1 designation.

The last subclass in this group contained a mix of 237 meningiomas with a heterogeneous spectrum of mutations outside of the genes mutated in other subclasses such as *NF2*, *TERT*p, *TP53*, *BAP1*, *PBRM1*, *AKT1*, *SMO* and *PIK3CA*. The majority of tumors in this subgroup occurred in women (n = 140, 59%). Chromosome 22q monosomy was detected in 112 cases (47.2%). The WHO grading was 88, 120 and 24 of grade 1, 2 and 3, respectively. This group will require further genomic characterization; some of the tumors in this class may ultimately be assigned to one of the other classes (for instance, 22q loss cases resolving to the *NF2*-associated canonical pathway above), whereas others may contain genomic or non-genomic drivers that are not yet associated with meningioma pathogenesis.

### Disseminated meningiomas

Our cohort includes genomic data from 35 (4%) metastatic meningiomas (21 females and 14 males) with 3 occurring as metastases in the spine and 32 occurring outside of the CNS. Extracranial systemic metastases (e.g., in lung, kidney, liver) were reported in 17 cases (49%), whereas the remaining cases disseminated to skin and spine. The majority of disseminated meningiomas were WHO grade 2 (n = 16) and grade 3 (n = 11). Metastatic meningiomas were observed across all three molecular subclasses. Alterations in *NF2* (n = 19, 54.3%), *CDKN2A* (n = 8, 22.9%), *BAP1* (n = 4, 11.4%), *ARID1A* (n = 4, 11.4%), *TERT*p (n = 3, 9.4%), and *TP53* (n = 2, 5.7%) were the most frequent relevant alterations detected in these cases. Interestingly, we did not observe significant association between any of these alterations with dissemination.

Finally, we did not detect recurrent clinically targetable gene rearrangements in our cohort (Additional file 1: Table S1).

## Discussion

This series of 850 meningiomas represents the largest collection of high-grade/progressive meningiomas with comprehensive genomic profiles presented to date. The samples originating from over 35 institutions were analyzed as part of a clinical care at Foundation Medicine. With the advantage of broad coverage of greater than 300 genes on our next-generation sequencing panel, the cohort is powered to examine clinical associations and gene–gene pathway interactions for a survey of the dominant pathways that contribute to the development of biologically-aggressive meningiomas. Taken together, we have identified at least three distinct molecular subclasses that define specific genomic tumor subgroups. Assigning meningiomas into distinct molecular subclasses will allow for a more detailed understanding of tumor-specific molecular features, for dissection of biological pathways, and for guiding approaches for diagnosis, treatment decisions and clinical trial design [17].

The first group, which we termed “canonical”, was the most commonly represented, and was associated with *NF2*-mutation and male-sex, and also harbored additional, previously described mutations in genes including *CDKN2A*/*B*, *KDM6A*, *ARID1A*, *PTEN*, *FBXW7*, and *SUFU* [2, 4, 20, 22, 30, 40]. Notably from a clinical standpoint, alterations of genes encoding regulators of the cyclin-dependent kinase (CDK) inhibitor pathway occurred in ~ 20% of *NF2*-mutant high-grade/progressive meningiomas. This frequent genomic finding is relevant because it indicates an opportunity for testing CDK4/CDK6 inhibitors in genomically-guided clinical trials (e.g., Alliance A071401 trial, NCT02523014). Furthermore, we found that the tumor suppressor gene and chromatin regulator *KDM6A*, which has been identified at low frequency in prior studies with smaller cohorts of meningioma, is more frequently altered in *NF2*-mutant high-grade meningiomas than previously noted [5, 20]. Indeed, previous work has observed that increased H3K27me3 levels and a hyper-methylated phenotype occupying the polycomb repressive complex (PRC2), is one of the hallmarks of *NF2*-mutant high-grade meningiomas [15, 22]. Taking into account that *KDM6A*, which is located on chromosome X and escapes X-inactivation, acts antagonistically to PRC2 and promotes H3K27 demethylation, our findings may partly explain the upregulated PRC2 activity in the *NF2*-mutant group of meningiomas after inactivation of *KDM6A* [10, 24, 38, 39].

The second group we identified contains both *NF2* mutant and wild-type tumors, and was not associated with either sex. We termed these poor prognosis meningiomas as “*NF2*-agnostic,” reflecting lack of associations with the presence or absence of *NF2* alterations in this cohort. *TERT*p mutations or *TP53* mutations typified this group, with recurrent mutations in the *TERT*p found in a substantial fraction of these meningiomas. In our large cohort, we estimate the frequency of *TERT*p mutations ranging from 2.5 to 14% in WHO grade 1 and WHO grade 3 meningiomas, respectively. These estimates must be tempered as the frequency of *TERT*p mutations might be underestimated in high-grade meningiomas due to intratumoral heterogeneity, the late emergence during tumor evolution, or the occurrence of other *TERT* alterations, such as gene rearrangements [15, 20, 21]. Some investigators have proposed a designation of “grade 4” meningioma for these meningiomas [25]. Interestingly, in our cohort, this group includes a relatively large subset of high-grade meningiomas that harbored *TP53* mutations, a gene alteration that has been previously seldom described in meningiomas or during meningioma progression [18, 20, 40]. This poor prognosis group may prove difficult to treat with targeted agents.

The final group is essentially exclusive of *NF2* mutations, containing at least three subgroups. These tumors are less frequent than canonical pathway lesions. The first subgroup is characterized by mutations in *BAP1* and/or *PBRM1*. Although *NF2*-wt cases have been less frequently linked with increased risk of progression, this *NF2*-wt subset is uniquely aggressive, which is captured within the current WHO classification that grades the majority of tumors in this subset as grade 3. Indeed, inactivation of the tumor suppressor gene *BAP1* has been linked to aggressive meningiomas with rhabdoid histo-morphology and was associated with a shorter progression-free survival [33, 34]. In addition, we have recently reported frequent *PBRM1* mutations in meningiomas with papillary histologic features [41]. *BAP1* and *PBRM1* inactivation can co-occur, and these tumors can have complex mixed histology patterns [33, 41]. Importantly, the frequent alterations of genes that encode epigenetic factors, such as the SWI/SNF complex genes *PBRM1* and *ARID1A*, in addition to *KDM6A*, *BAP1* and *FBXW7* indicate that dysregulation of chromatin remodeling is a common feature across all higher-grade meningiomas, regardless of the molecular subgroup [2, 20, 24, 33, 34, 39].

An additional subgroup of the *NF2*-exclusive category consists of the previously described subset of skull base meningiomas with *AKT1*, *SMO* or *PIK3CA* mutations and predominantly WHO 1 grading [1, 5, 6, 31]. It is important to note that tumors with these mutations are generally grade 1 but their presence in higher grade and progressive meningiomas suggests that they are not exclusively indicators of non-aggressive behavior. This may, in part, be due to the confined anatomic location of these tumors.

In contrast to this subgroup, the last subgroup in the *NF2*-exclusive category included meningiomas with unknown drivers and with heterogeneous molecular mutations that lacked *NF2*, *TERT*p, *TP53*, *BAP1*, *PBRM1*, *AKT1*, *SMO* and *PIK3CA* mutations. In half of the cases in this subgroup, chromosome 22 monosomy was detected and was associated with a higher WHO grade. It is possible that some of these lesions may contain cryptic *NF2* inactivation that was not detected with our sequencing methods. Nevertheless, further genomic large-scale analyses are needed to collect more genomic information and accurately subdivide the latter subgroup.

Interestingly, we did not detect frequent clinically targetable gene rearrangements in any of the subclasses in our study, indicating that progressive/high-grade meningiomas are seldom driven by gene rearrangements that are characterized in our genomic assay and are ineligible for targeted therapeutic agents against fusion oncogenes (e.g., NTRK, ALK, etc.). Moreover, none of the cases in our series harbored hotspot *IDH1* or *IDH2* mutations, which contrasts with recent reports [13, 29].

Finally, we identified and genotyped 35 disseminated meningiomas, with half of the cases with metastases outside of the CNS. Our study adds to the existing literature by providing genomic data on metastatic meningiomas which are quite rare (2%) [9]. We show that metastatic meningiomas are derived from all three subclasses in our study, with half of cases harboring *NF2*-mutations. Interestingly, loss of merlin, the protein encoded by *NF2*, has been linked to higher cell motility and tumor invasion [23, 35]. However, further functional studies are needed to explore the role of *NF2* loss in tumor dissemination. One limitation of our study is the lack of treatment and survival data, which prevents comparison of the outcomes between the different molecular groups. In addition, although our NGS panel enables a broad survey of known cancer genes, it does not screen for relevant genomic alterations such as *DMD* deletions or *TERT* gene translocations both of which have been shown to be associated with a poor outcome in progressive/high-grade meningiomas [20]. Therefore, further studies with whole exome sequencing are warranted to confirm and extend our findings. Future studies will additionally need to address intra-tumor heterogeneity, since our sub-classification of meningiomas does not capture the evolving landscape of intra-tumor heterogeneity and evolutionary progression in high-grade/progressive meningiomas. In addition, comparison to newly established DNA methylation-based classifications and to studies of genome-wide copy number alterations has the potential to provide further important insights into biologically-aggressive meningiomas. Previous studies have developed and validated models using DNA methylation-based arrays to provide important prognostic information to guide therapeutic interventions in meningioma patients [26, 32].

In summary, our analysis identified at least three distinct genomic groupings within high-grade/progressive meningiomas, findings that can outline a genotype-driven sub-typing of these tumors. As with medulloblastomas, gliomas and other CNS tumors that are now defined by molecular parameters in addition to histology, we envision that such findings will aid in the transition towards a similarly integrated diagnostic approach for the molecular subclassification of meningiomas. In addition, we anticipate that molecular subclasses of aggressive meningiomas will have significant implications for the design of therapeutic trials in this patient population.

## Supplementary information

**Additional file 1: Supp. Table 1:** Genomic data with all detected single nucleotide variants, small insertions/deletions, and chromosome arm data. The samples were assayed by adaptor ligation hybrid capture, performed for all coding exons of 236 (v1), 315 (v2), or 405 (v3) cancer-related genes plus select introns from 19 (v1), 28 (v2), or 31 (v3) genes frequently rearranged in cancer.

**Additional file 2: Figure S1** shows the genome-wide copy-number alteration data of eligible cases. Loss of chromosome 22q (84.1%) and loss of chromosome 1p (68.8%) were the most common copy number alterations.

**Additional file 3: Figure S2:** a stacked bar graph demonstrates the most frequently detected mutations in meningiomas, based on their WHO grading. In 13 cases, WHO grading was not available. Those cases included: four meningiomas with NF2 mutations and alterations in PTEN (n= 1), ARID1A (n= 1), BAP1 (n= 1) and PTEN (n= 1).
