# Supplementary figures and images for: Distinct genomic subclasses of high-grade/progressive meningiomas: NF2-associated, NF2-exclusive, and NF2-agnostic
Source: Acta Neuropathol Commun. 2020 Oct 21;8:171. doi: 10.1186/s40478-020-01040-2 (PMC7580027; doi:10.1186/s40478-020-01040-2)

Aneuploidy   Gain   Loss   None

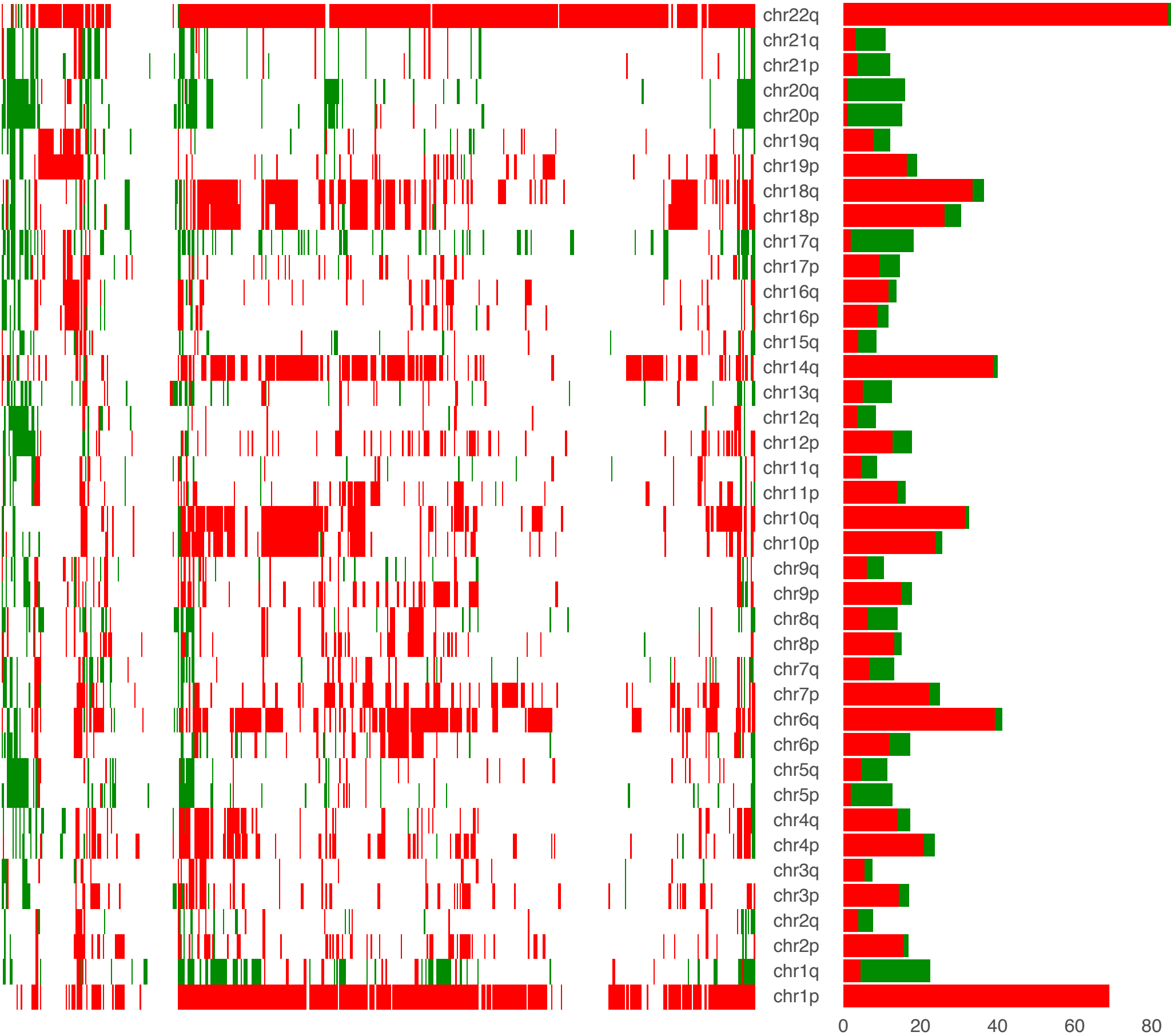

Percentage of samples

Supplement: Supplementary file 2 — Additional file 2: Figure S1 shows the genome-wide copy-number alteration data of eligible cases. Loss of chromosome 22q (84.1%) and loss of chromosome 1p (68.8%) were the most common copy number alterations. [file 40478_2020_1040_MOESM2_ESM.pdf]
