# Supplementary material for: Distinct genomic subclasses of high-grade/progressive meningiomas: NF2-associated, NF2-exclusive, and NF2-agnostic
Source: Acta Neuropathol Commun. 2020 Oct 21;8:171. doi: 10.1186/s40478-020-01040-2 (PMC7580027; doi:10.1186/s40478-020-01040-2)
Supplement: Supplementary file 3 — Additional file 3: Figure S2: a stacked bar graph demonstrates the most frequently detected mutations in meningiomas, based on their WHO grading. In 13 cases, WHO grading was not available. Those cases included: four meningiomas with NF2 mutations and alterations in PTEN (n= 1), ARID1A (n= 1), BAP1 (n= 1) and PTEN (n= 1). [file 40478_2020_1040_MOESM3_ESM.docx]

In 13 cases, WHO grading was not available. Those cases included: four meningiomas with *NF2* mutations and alterations in *PTEN* (n= 1), *ARID1A* (n= 1), *BAP1* (n= 1) and *PTEN* (n= 1).
